# A lysing polysaccharide monooxygenase from *Aspergillus niger* effectively facilitated rumen microbial fermentation of rice straw

**DOI:** 10.5713/ab.24.0026

**Published:** 2024-05-07

**Authors:** Ting Li, Kehui OuYang, Qinghua Qiu, Xianghui Zhao, Chanjuan Liu

**Affiliations:** 1 Jiangxi Province Key Laboratory of Animal Nutrition/Engineering Research Center of Feed Development, Jiangxi Agricultural University, Nanchang Jiangxi 330045, China

**Keywords:** *In vitro* Rumen Fermentation, Lysing Polysaccharide Monooxygenase, Microbial Community, Rice Straw, Surface Structure

## Abstract

**Objective:**

This study investigated the impact of *Aspergillus niger* lysing polysaccharide monooxygenase (*An*LPMO) on *in vitro* rumen microbial fermentation of rice straw.

**Methods:**

*An*LPMO was heterologously expressed in *Escherichia coli*. Fourier transform infrared spectrometry and X-ray photoelectron spectroscopy analyzed the surface structure of rice straw after *An*LPMO treatment. Two *in vitro* experiments, coupled with 16S high-throughput sequencing and quantitative real–time polymerase chain reaction techniques, assessed the influence of *An*LPMO on rumen microbial fermentation of rice straw.

**Results:**

*An*LPMO exhibited peak activity at 40°C and pH 6.5, with a preference for rice straw xylan hydrolysis, followed by Avicel. *An*LPMO application led to the fractional removal of cellulose and hemicelluloses and a notable reduction in the levels of carbon elements and C–C groups present on the surface of rice straw. Compared to the control (no *An*LPMO), supplementing *An*LPMO at 1.1 to 2.0 U significantly enhanced *in vitro* digestibility of dry matter (IVDMD, p<0.01), total gas production (p<0.01), and concentrations of total volatile fatty acids (VFA, p<0.01), acetate (p<0.01), and ammonia-N (p<0.01). Particularly, the 1.4 U *An*LPMO group showed a 14.8% increase in IVDMD. In the second experiment, compared to deactivated *An*LPMO (1.4 U), supplementing bioactive *An*LPMO at 1.4 U increased IVDMD (p = 0.01), total gas production (p = 0.04), and concentrations of total VFA (p<0.01), propionate (p<0.01), and ammonia-N (p<0.01), with a limited 9.6% increase in IVDMD. Supplementing *An*LPMO stimulated the growth of ruminal bacterial taxa facilitating fiber degradation, including Proteobacteria, Spirochaetes, *Succinivibrio*, *Rikenellaceae_RC9_Gut_Group*, *Prevotelaceae_UCG-003*, *Desulfovibrio*, *Fibrobacter succinogenes*, *Ruminococcus albus*, *R. flavefaciens*, *Prevotella bryantii*, *P. ruminicola*, and *Treponema bryantii*.

**Conclusion:**

These findings highlight *An*LPMO’s potential as a feed additive for improving rice straw utilization in ruminant production.

## INTRODUCTION

Rice straw, a global byproduct of rice farming, is valued for its substantial fibrous carbohydrate content, with 33% to 47% cellulose and 19% to 27% hemicellulose, making it a common forage alternative for ruminants [1]. However, its digestibility typically falls below 50%, despite the presence of a diverse range of rumen microorganisms proficient in rice straw digestion [2]. This mainly is attributed to two factors [3]: the intricate matrix of cellulose, hemicellulose, and lignin in the cell wall that restricts microbial and enzymatic access to fiber interiors, and the highly ordered crystalline structure of cellulose that hinders efficient breakdown by rumen microorganisms. As a result, rice straw is often discarded, contributing to environmental pollution through decay or burning. Physical and chemical treatments like steam explosion and chemical alkaline or acidic hydrolysis have been effective in enhancing fiber accessibility and digestibility by disrupting lignin-carbohydrate complexes and cellulose crystallinity [3,4]. However, these methods are hampered by high costs, energy intensiveness, and potential risks to animals and the environment, particularly with alkaline treatments [4]. In contrast, enzymatic catabolism effectively simplifies complex substances into their basic units, demonstrating notable efficacy and specificity under mild conditions, which has sparked significant research interest for crop straw processing and utilization.

Lytic polysaccharide monooxygenases (LPMOs), copper-dependent enzymes discovered in 2010, cleave polysaccharides through oxidative cleavage [5]. The unique and most attractive aspect of some LPMOs is their ability to attack polysaccharides with recalcitrant structures, such as crystalline cellulose, chitin, and cellulose-hemicellulose complexes and create chain ends tractable by canonical glycoside hydrolases (GHs) for further depolymerisation [5,6], which makes it display a high synergistic effect with fibrolytic enzymes or other polysaccharases [7]. For example, a LPMO from *Myceliophthora thermophila* C1 showed a strong synergistic effect with endoglucanase I with a 8-fold and 16-fold higher release of detected oligosaccharides from regenerated amorphous cellulose, respectively, compared to the oligosaccharides release of LPMO and endoglucanase I alone [6]. LPMOs’ exceptional characteristics have spurred extensive investigation into their use for lignocellulose saccharification [8]. However, their impact on ruminal fiber degradation and forage utilization in ruminants remains unexplored.

The rumen harbors a diverse assemblage of anaerobic microorganisms, including bacteria, archaea, and eukaryotes, that collectively produce a broad range of lingo-cellulolytic enzymes, which means that the rumen serves as a conducive milieu for LMPO, offering an enzyme-abundant setting to enhance its catalytic efficacy. Consequently, we hypothesize that supplementing the rumen with LPMO could enhance digestion and fermentation of fibrous feed. For this purpose, this study cloned and heterologously expressed an AA9 LPMO from *Aspergillus niger* (*An*LPMO) by *Escherichia coli* (*E. coli*) and investigated its characteristics and effects on surface structure and *in vitro* ruminal fermentation of rice straw. This study set out to explore a new approach for improving ruminal fiber degradation, which will provide important significance in theory and practice for promoting efficient utilization of fibrous roughage and ruminant production.

## MATERIALS AND METHODS

### Cloning of *AnLPMO* gene and construction of expression vector

*A. niger* was used in the current study was purchased from China General Microbiological Culture Collection Center (Number: CGMCC 3.17612). The *AnLPMO* gene was cloned by extracting total RNA from *A. niger* that had been cultivated for a duration of 2 d in a culture medium. Subsequently, cDNA synthesis was performed using the EasyScript One-Step gDNA Removal and cDNA Synthesis SuperMix kit (AE311-02; TransGen Biotech, Beijing, China). The *AnLPMO* gene was amplified by polymerase chain reaction (PCR) using primers *AnLPMO*-F (AAGAAGGAGATATACGAA TTCATGAAGACTACCACCTACAGTT) and *AnLPMO*-R (GTGGTGGTGGTGGTGGTGCTCGAGCTGAGACGC AACGCACTGGTAG) to the pET-28a vector according to genome data of *A. niger* (GenBank accession number: CAK 42466.1). The PCR products were ligated into the pET-28a vector, specifically designated pET-*An*LPMO, and introduced into *E. coli* DH5α cells through the heat shock method. The pET-*An*LPMO plasmids were extracted, confirmed through PCR amplification, and subjected to sequencing analysis.

### Expression and purification of *An*LPMO

The plasmid pET-*An*LPMO was introduced into competent *E. coli* BL21 (DE3) cells through transformation. The positive transformants were screened by PCR. Then the induction expression, analysis, and purification of *An*LPMO were carried out in accordance with our previous methodology using isopropyl-β-D-thiogalactoside (IPTG), sodium dodecyl sulfate polyacrylamide gel electrophoresis (SDS-PAGE), western blotting, and Ni-charged affinity chromatography [9].

### Characterization of *An*LPMO

The optimal pH of *An*LPMO was analyzed in 0.05 M citrate-phosphate buffer (pH 3.0 to 8.0) at 37°C for 6 h with 1% Avicel PH-101 as a substrate. The quantification of released reducing sugars was conducted utilizing an alkaline 3,5-dinitrosalicylic acid reagent. Likewise, the investigation into the temperature dependence of *An*LPMO was carried out by subjecting reaction mixtures to incubation within the range of 30°C to 55°C, while maintaining the optimal pH. One unit of enzyme activity (U) was defined as the amount of enzyme leading to the production of 1 μmol of glucose per h at the optimal pH and temperature. The determination of the substrate specificity of *An*LPMO was conducted using a reaction mixture comprising 1% of the desired substrates in a 0.05 M sodium citrate buffer, as described previously. The substrates included rice straw xylan, sodium carboxymethylcellulose, Avicel, chitosan, and rice straw.

### Structural analysis of rice straw

A 5 mL mixture containing 0.3 U purified *An*LPMO and 50 mg rice straw in the 0.05 M sodium citrate buffer (pH 6.5) were incubated and shaken for 6 h at 40°C. The control without *An*LPMO addition was incubated simultaneously. After incubation, the reaction mixture was centrifuged at 12,000 rpm for 2 min, and the supernatant was discarded. The precipitate was dried at 65°C for 48 h for structural analysis of rice straw by Fourier transform infrared spectrometry (FTIR) and X-ray photoelectron spectroscopy (XPS) according to our previous description [10]. All incubations were done in triplicate. All samples from both the control group and the *An*LPMO group were subjected to XPS analysis. Three samples were combined individually from both the control group and the *An*LPMO group to form a single sample for FTIR analysis. The XPS data were processed using the Thermo Avantage ver. 5.9931 software.

### *In vitro* rumen fermentation of rice straw

An *in vitro* experiment (Experiment 1) was performed to investigate the effects of *An*LPMO on rumen fermentation of rice straw. This study was approved by the Animal Care and Use Committee of the Jiangxi Agricultural University (JXAULL-2023-10-19). In short, rumen liquid was obtained from three ruminally fistulated beef cattle, subsequently filtered through four layers of cheesecloth, and combined in a 1:2 ratio (v/v) with anaerobic buffer. All procedures were conducted under anaerobic conditions, employing continuous CO_2_ flushing. The fermentation was conducted in 120-mL serum bottles containing 600 mg of rice straws, 0 to 2.0 U purified *An*LPMO and 60 mL of buffered rumen fluid. The bottles were hermetically sealed and placed in a water bath with agitation at 39°C for 48 h. Incubation of all samples was done in triplicate. The gas production during the fermentation process was estimated using a 100 mL glass syringe connected to the fermentation bottle [11]. The fermentation process was halted by subjecting the bottles to a cooling environment using ice, and a pre-weighted filter crucible was used for filtering the residue to determine the *in vitro* digestibility of dry matter (IVDMD). The pH of filtrate was determined immediately. One milliliter of filtrate was stored by mixing with a deproteinizing solution (100 g/L metaphosphoric acid and 0.06 g/L crotonic acid) in equal proportion for the determination of volatile fatty acids (VFA). The remaining filtrate was preserved to determine ammonia-N concentration. Experimental treatments were evaluated in two incubation runs.

In order to rule out the possible interference caused by the increase in nitrogen supply to microorganisms by supplementing *An*LPMO, and to verify the reproducibility of the above results, a second *in vitro* rumen fermentation was conducted (Experiment 2). The basic operation process was the same as the above description. AnLPMO (1.4 U) was added into the incubation bottles as treatments, and the same amounts of heat-inactivated *An*LPMO (boiling in water for 10 min) were incubated similarly as controls. The collected filtrate was also used for DNA extraction and microbial community determination in addition to the analysis of pH, VFA, ammonia nitrogen according to the previous description.

### Analytical procedures

The determination of DM content in the samples was conducted through a drying process at 65°C for 72 h. The analysis of ammonia-N in the samples followed the methodology outlined by Weatherburn [12]. The VFA in the samples were determined using a high-performance liquid chromatography (HPLC) system (model D-7000; Hitachi Ltd., Tokyo, Japan) connected to an Agilent Eclipse XDB-C18 column (4.6×250 mm, 5 μm). Crotonic acid served as the internal standard. Microbial genomic DNA was extracted from fermentation liquid using the E.Z.N.A. stool DNA Kit (Omega Bio-tek, Norcross, GA, USA). The V3–V4 region of 16S rDNA was amplified by PCR and was analyzed by Guangdong Magigene Biotechnology Co., Ltd. (Guangzhou, China). The Illumina Nova 6000 platform was used for PE250 sequencing of library. The estimation of bacterial richness indices was conducted through the Ace and Chao method, while the determination of bacterial diversity indices was accomplished using the Simpson and Shannon method. Principal coordinate analysis (PCoA) was utilized to generate two-dimensional plots by employing the Bray-Curtis distances. The linear discriminant analysis (LDA) effect size (LEfSe) method was employed to ascertain the taxonomic entities that exhibited the greatest differential abundance between groups with an absolute LDA score ≥3.0. The co-occurrence network among bacteria genera was built based on Pearson correlation coefficients and p-values. The cutoff of correlation coefficients and p-values was determined as 0.80 and 0.05, respectively. The correlation between bacteria genera and fermentation parameters was analyzed by Redundancy analysis (RDA) method. The bacteria genera were also tested for Pearson correlation calculations with ruminal fermentation parameters and a heat map was generated. The raw sequencing data have been deposited in the National Center for Biotechnology Information under accession number PRJNA1036034. Relative quantification of some fibrolytic bacteria and non-fibrolytic bacteria was performed according to previously reported methods and primers [13,14].

### Statistical analyses

Data in Experiment 1 were analyzed by the general linear model (univariate) using IBM SPSS statistics version 20 (IBM, Chicago, IL, USA) according to *Y_ij_* = *μ*+*A_i_*+*B_j_*+*ɛ_ij_*, where *Y_ij_* is the dependent variable; *μ* is the overall mean; *A_i_* is the effect of the dietary treatment; *B_j_* is the random effect of incubation runs; *ɛ_ij_* the residual error. Other data was analyzed by the independent-samples t-test using SPSS. Significance was declared at p≤0.05. The multiple comparisons were carried out by the least significant difference test.

## RESULTS

### Cloning and expression of *An*LPMO

**Figure 1:**
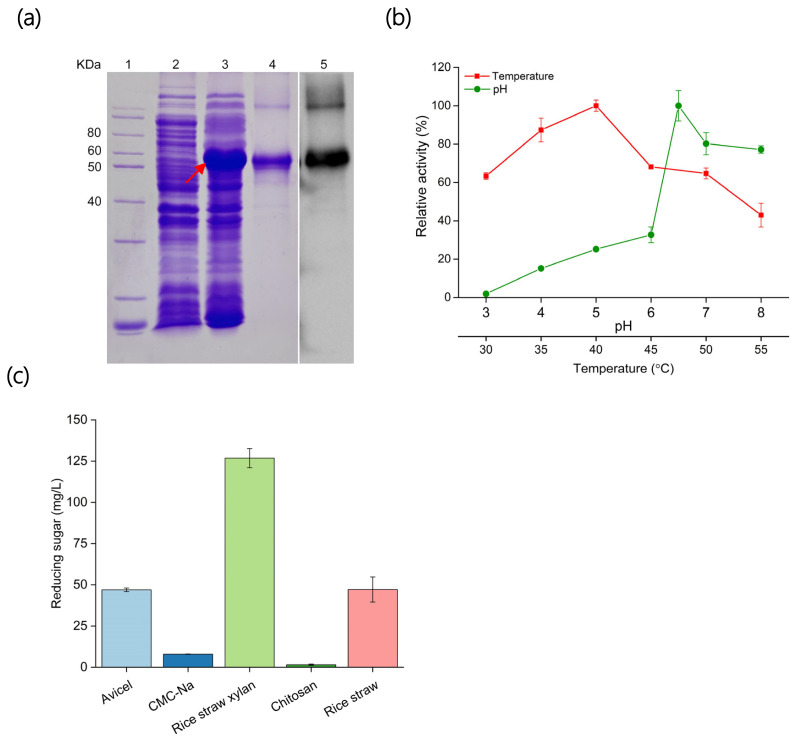
Heterologous expression and characteristic analysis of *An*LPMO. (a) Analysis of *An*LPMO by SDS-PAGE and Western blot: 1, protein marker; 2, non-transformed *E. coli* BL21 (DE3); 3, *An*LPMO transformants induced with 0.5 mM IPTG; 4, purified *An*LPMO from supernatants obtained after ultrasonication of *An*LPMO transformants; 5, Western blot analysis of purified *An*LPMO; The red arrow indicates the expression of *An*LPMO. (b) Determination of the optimal pH and temperature for *An*LPMO. (c) The substrate specificity of *An*LPMO. *An*LPMO, *Aspergillus niger* lysing polysaccharide monooxygenase; SDS-PAGE, sodium dodecyl sulfate polyacrylamide gel electrophoresis; IPTG, isopropyl-β-D-thiogalactoside.

The *An*LPMO-encoding gene was amplified from *A. niger* genomic cDNA and the DNA fragment obtained was sequenced. The *AnLPMO* gene exhibited a high similarity with the reference sequence from genome data of *A. niger* (GenBank accession number: CAK42466.1) 98.6%, whereas the amino acid sequence similarity was 99.3% with 8 different amino acids between them (Supplementary Figure S1). Expressed and purified *An*LPMO was analysed and verified by SDS-PAGE and Western blot (Figure 1a). The *An*LPMO protein was detected as a single band at molecular mass 51 kDa.

### Characteristics of *An*LPMO

The *An*LPMO demonstrated peak activity at pH 6.5, with approximately 80% of its maximum activity observed within the pH range of 7.0 to 8.0 (Figure 1b). However, a notable decrease in *An*LPMO activity was observed when the pH dropped below 6.0, leading to less than 40% of its maximum activity. *An*LPMO had the optimal activity at 40°C and maintained over 60% of its maximum activity between 30°C to 50°C (Figure 1b). The substrate specificity results showed that *An*LPMO exhibited a pronounced preference towards rice straw xylan, moderate preference towards Avicel and rice straw, and feeble activity towards CMC-Na and chitosan (Figure 1c).

### Surface characterization of rice straw

**Figure 2:**
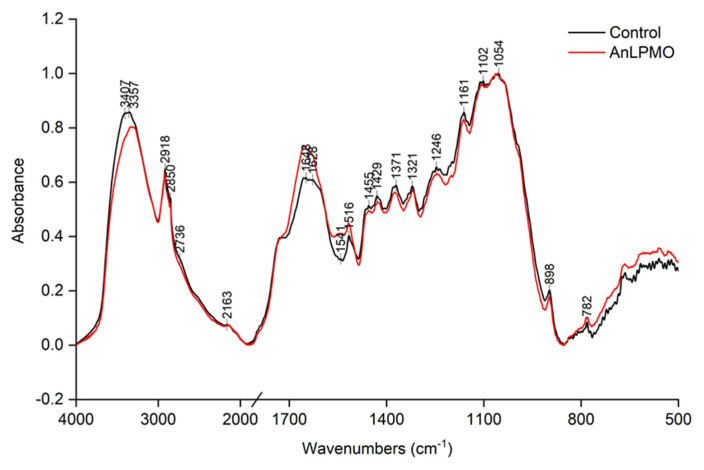
FTIR spectra of control and *An*LPMO-treated rice straws. FTIR, Fourier transform infrared spectrometry; *An*LPMO, *Aspergillus niger* lysing polysaccharide monooxygenase.

Figure 2 illustrates the FTIR spectra of rice straw treated with/without *An*LPMO. Significant disparities were observed in the band spectrum of the two groups. In the control group, some pronounced peaks at approximately 1,161, 1,246, 1,371, 1,429, 1,455, and 3,357 cm^−1^ were observed, whereas the intensity of these peaks was diminished in the rice straw treated with *An*LPMO. Contrarily, the presence of *An*LPMO resulted in an amplification of the peak intensity around 1,516, 1,545, 1,628, and 1,648 cm^−1^.

**Figure 3:**
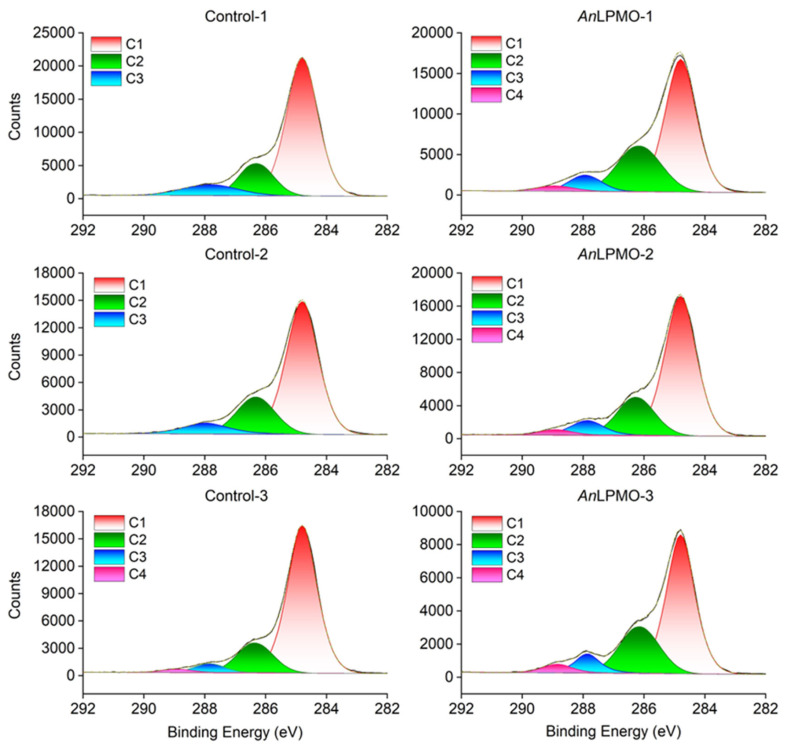
High-resolution deconvoluted C1s spectra of control and *An*LPMO-treated rice straws. Contron-1, Control-2, and Control-3: three biological duplicate samples for control group; *An*LPMO-1, *An*LPMO-2, and *An*LPMO-3: three biological duplicate samples for *An*LPMO group. *An*LPMO, *Aspergillus niger* lysing polysaccharide monooxygenase.

The results obtained from the XPS analysis are presented in Table 1 and Figure 3. The presence of the elements carbon, oxygen, nitrogen, and silicon was detected in all of the samples. The *An*LPMO caused a decrease in the carbon (p< 0.01) and silicon (p<0.01) atomic composition on the surface of rice straw, accompanied by a concomitant increase in nitrogen atoms content (p<0.01). There was a lack of statistically significant disparity observed in the levels of oxygen atoms proportion and O/C ratio between the two groups. In the present study, the high-resolution C1s spectra were divided into four distinct subpeaks, namely C1 (C-C/C-H), C2 (C-O/C-N), C3 (C=O/O-C-O), and C4 (O=C-O). The proportion of C1 (p = 0.05) decreased with modification of rice straw by *An*LPMO, while an increase in amount of C2 (p = 0.09) and C4 (p = 0.02) was observed.

### *In vitro* fermentation of rice straw

Table 2 presents the impact of various doses of *An*LPMO on rumen fermentation parameters of rice straw. In comparison to the control group, the addition of *An*LPMO at a dosage range of 0.8 to 2.0 U resulted in a significant enhancement of IVDMD (p<0.01). The most substantial improvement was observed in the 1.4 U group, where an increase of 14.8% was recorded. The introduction of *An*LPMO resulted in a significant augmentation of total gas production (p<0.01), as evidenced by a notable increase of 9.89 mL in the 2.0 U group when compared to the control group. A significant increase in the concentration of total VFA (p<0.01) and acetate (p<0.01) was observed when the supply of *An*LPMO was elevated to 1.1 to 2.0 U relative to the control group. The addition of *An*LPMO resulted in a higher level of propionate concentration (p<0.01). However, it was only when the *An*LPMO dosage was increased to 2.0 U that the propionate production reached a significantly higher level compared to other *An*LPMO groups. Supplying *An*LPMO reduced butyrate concentration and the ratio of acetate and propionate relative to the control group (p<0.05). The incremental increase in ammonia-N concentration was observed as the *An*LPMO added was augmented (p<0.01). In particular, upon the addition of 2 U of *An*LPMO, there was an observed increase in ammonia-N concentration by 10.04 m*M*.

To eliminate the potential impact of *An*LPMO as nitrogen source, the control group was supplemented with deactivated *An*LPMO to investigate the impact of *An*LPMO activity on the ruminal fermentation of rice straw. The presence of *An*LPMO resulted in a substantial increase in IVDMD (p = 0.01), total gas production (p = 0.04), and the concentration of total VFA (p<0.01), propionate (p<0.01), and ammonia-N (p<0.01) relative to the control group (Table 3). Specifically, the increase in IVDMD and total gas production amounted to 9.6% and 16 mL, respectively. Additionally, there was a tendency for an increase in acetate concentration.

### Microbial community during *in vitro* fermentation of rice straw

**Figure 4:**
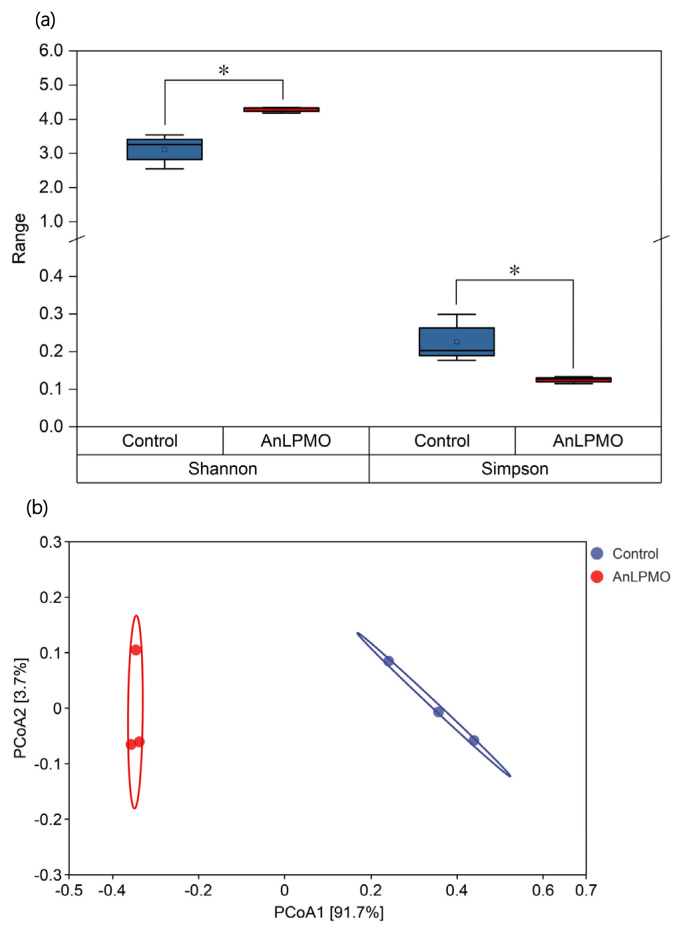
Alpha diversity (a) and beta diversity (b) of rumen bacteria in samples. * p<0.05.

The effects of *An*LPMO on the microbial community in *in vitro* ruminal fermentation of rice straw were investigated using high-throughput sequencing technology. A sum of 753,468 clean reads was obtained from the six samples. These reads were assigned to 787 operational taxonomic units (OTUs) according to the 97% sequence identity. The estimation of bacterial richness indices was conducted using the Ace and Chao method, while the determination of bacterial diversity indices was performed utilizing the Simpson and Shannon method, based on the OTUs. The statistical analysis revealed that there were no statistically significant disparities in the Ace and Chao indices between the control group and the *An*LPMO group (Supplementary Table S1). However, the control group had higher Simpson index (p = 0.05) and lower Shannon index (p = 0.02) compared to the *An*LPMO group (Figure 4a). PCoA was conducted using the Bray-Curtis metric to investigate the dissimilarities in microbial composition between the control and *An*LPMO groups. The findings indicated that the microbiota exhibited distinct clustering patterns, with the axes explaining 95.3% of the total observed variation (Figure 4b). This suggested that specific bacterial species may serve as defining characteristics for the microbiota of *An*LPMO groups.

**Figure 5:**
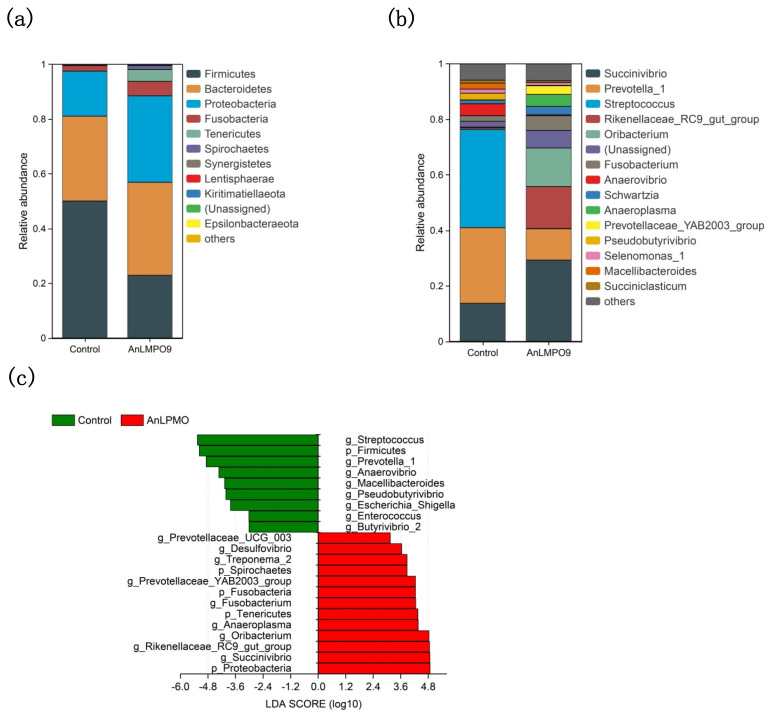
Composition and distribution of rumen bacteria in samples. (a) and (b), The relative abundance of top 15 abundant taxa of samples in the phylum level and genus level, respectively; (c), LEfSe analysis displaying the ruminal bacteria change in the phylum level and genus level between control group and *An*LPMO group (LDA≥3.0 and p≤0.05 were shown). *An*LPMO, *Aspergillus niger* lysing polysaccharide monooxygenase.

A total of 15 phyla and 91 genera were identified in this study. There were observed variations in the phylum compositions between the two groups (Figure 5a). The top three bacterial phyla in the control group were Firmicutes (50.1%), Bacteroidetes (31.0%), and Proteobacteria (16.4%), while in the *An*LPMO group were Bacteroidetes (34.0%), Proteobacteria (31.7%), and Firmicutes (22.8%). At the genus level, *Streptococcus* (35.4%), *Prevotella_1* (27.2%), and *Succinivibrio* (13.7%) were the dominant bacteria in the control group, but *Succinivibrio* (29.3%), *Rikenellaceae_RC9_gut_group* (15.1%), and *Oribacterium* (13.9%) were the dominant bacteria in the *An*LPMO group (Figure 5b).

In order to ascertain the taxon distributions, LEfSe analysis was conducted, leading to the discovery of biomarkers in both the *An*LPMO and control groups. There were 66 taxa found as biomarkers (Supplementary Figure S2). At the phylum level, the presence of *An*LPMO resulted in an increase in four phyla, namely Proteobacteria, Tenericutes, Fusobacteria, and Spirochaetes, while Firmicutes experienced a reduction (Figure 5c). At the genus level, 9 taxa were increased by *An*LPMO, among which *Succinivibrio*, *Rikenellaceae_RC9_gut_group*, and *Oribacterium* were found to be predominant bacteria, and 8 taxa were reduced, among which *Streptococcus*, *Prevotella_1*, and *Anaerovibrio* were predominant bacteria.

In this study, the relative abundance of some fibrolytic bacteria and non-fibrolytic bacteria at the species level was quantified by quantitative real–time polymerase chain reaction (qRT-PCR). Supplemental *An*LPMO resulted in a notable increase in the relative abundance of three typical fibrolytic bacteria, namely, *Fibrobacter succinogenes*, *Ruminococcus albus*, and *R. flavefaciens* (Table 4). Furthermore, the *An*LPMO exhibited a substantial enhancement in the relative abundance of *Prevotella bryantii*, *P. ruminicola*, and *Treponema bryantii*, with a particularly notable increase observed in *P. bryantii* and *T. bryantii*, which experienced a remarkable surge of approximately 5,700 and 800 folds, respectively, when compared to the control group. In contrast, supplemental *An*LPMO resulted in approximately 10-fold and 5300-fold reductions in the relative abundance of *Anaerovibrio lipolytica* and *Streptococcus bovis*, respectively.

Co-occurrence network analysis was employed to examine the correlation among the top 30 most abundant bacteria at the genus level. The co-occurrence network that emerged consisted of a total of 26 nodes and 152 edges (Supplementary Figure S3). *Anaerovibrio*, *Prevotellaceae_UCG-003*, *Rikenellaceae_RC9_gut_group*, *Streptococcus*, and *Succinivibrio* exhibit a higher number of edges, each surpassing the threshold of 10 edges. *Succinivibrio*, *Rikenellaceae_RC9_gut_group*, and *Oribacterium* were the dominant bacteria within the *An*LPMO group. *Succinivibrio* exhibited positive correlations with *Rikenellaceae_RC9_gut_group*, *Prevotellaceae_Ga6A1_Group*, *Desulfovibrio*, *Fuscobacterium*, *Schwartzia*, and *Prevotellaceae_UCG_003*, while displaying negative correlations with *Psedobutyrivibrio*, *Escherichia-Shigella*, *Streptococcus*, *Anaerovibrio*, and *Prevotella_1*. *Streptococcus*, the genus exhibiting the highest relative abundance within the control group, demonstrated a negative correlation with *Succinivibrio*, *Desulfovibrio*, *Prevotellaceae_Ga6A1_Group*, *Oribacterium*, *Rikenellaceae_RC9_gut_group*, *Fusobacterium*, *Prevotellaceae_UCG-003*, and *Lachnospiraceae_FCS020*, while displaying a positive correlation with *Enterococcus*, *Anaerovibrio*, *Prevotella_1*, and *Pseudobutyrivibrio*, which served as biomarkers within the control group.

The correlation between bacteria at genus level and fermentation parameters was analyzed. The RDA results showed that the bacteria composition of samples in the control group was clearly separated from those in the *An*LPMO group at the first constrained axis. Genera enriched in *An*LPMO group were positively correlated with acetate, propionate, total VFA, IVDMD, and ammonia-N (Supplementary Figure S4a). To further investigate the connection between ruminal fermentation and the microbial community, the correlation matrices were generated based on the relative abundance of microbiota and the *in vitro* fermentation parameters (Supplementary Figure S4b). IVDMD and the concentrations of ammonia-N, acetate and total VFA exhibited positive correlations with *Rikenellaceae_RC9_gut_group*, *Succinivibrio*, *Desulfovibrio*, and *Prevotellaceae_UCG-003*. Conversely, these factors displayed negative correlations with *Streptococcus*. The concentration of propionate exhibited positive correlations with *Succinivibrio*, *Fusobacterium*, *Prevotellaceae_Ga6A1_group*, and *Schwartzia*, while displaying negative correlations with *Pseudobutyrivibrio*, *Escherichia-Shigella*, *Prevotella_1*, *Streptococcus*, and *Anaerovibrio*. Additionally, *Oribacterium* demonstrated positive correlations with IVDMD and ammonia-N concentration, whereas *Treponema_2* exhibited positive correlations with acetate and total VFA.

## DISCUSSION

The enhanced hydrolysis efficiency of cellulolytic enzymes through the synergistic effect of LPMOs has been extensively demonstrated [6,7]. However, the potential impact of LPMOs on rumen fermentation of forage, in conjunction with rumen microbial cellulolytic enzymes, remains unexplored in existing literature. Hence, the primary focus of this study was to examine the impact of *An*LPMO on *in vitro* rumen fermentation and microbial communities associated with rice straw. To our knowledge, this is the first report about the connection between LPMOs and ruminal fermentation.

This study employed FTIR and XPS techniques to examine the impacts of *An*LPMO on the surface structure of rice straw. The FTIR peaks at 3,407, 1,371, 1,429, and 1,455 cm^−1^ correspond to −OH stretching, C-H deformation vibration, O-H in plane bending of alcohol groups, and asymmetric C-H bending from methoxyl groups, respectively, for cellulose [15]. These peaks’ intensity experienced a noticeable decrease subsequent to the *An*LPMO treatment, indicating the degradation of the cellulose structure in rice straw. The bands at 1,516 and 1,648 cm^−1^ were usually attributed to structural specifications of lignin [15]. It was clear that both bands had greater intensity compared to that in the untreated rice straw. This heightened intensity can be attributed to the degradation of cellulose components, resulting in a corresponding augmentation in the lignin content within the rice straw. The hemicellulose-related characteristic peaks were observed at approximately 1,246 cm^−1^, which was due to the stretching of C-O [16]. The *An*LPMO-treated substrates showed lower absorbed intensity at 1,246 cm^−1^, indicating the potential removal of xylan from rice straw by *An*LPMO.

*An*LPMO significantly decreased the carbon atom composition on the surface of rice straw, thereby providing further evidence of *An*LPMO’s capability to degrade fibrous materials present in rice straw. The nitrogen atom concentration on the surface of rice straw treated with *An*LPMO exhibited a substantial increase in response. It had been proposed that the cell wall protein is connected to the polysaccharide via isotyrosine and diisotyrosine bridges [17]. The relocation of the component to the biomass surface following the cleavage of the lignin-carbohydrate complex during enzymatic hydrolysis may lead to an augmentation in the nitrogen signals [17]. C1s can be deconvoluted into four Gaussian peaks, which were found to be at about 284.8 eV, 286.3 eV, 287.9 eV, and 289.0 eV in the present study, corresponding to C1 (C-C/C-H), C2 (C-O/C-N), C3 (C=O/O-C-O), and C4 (O=C-O) groups, respectively [18,19]. The displacement or intensity changes of the peaks serve as indicators of variations in the chemical structures of the samples. The proportion of C1 decreased with *An*LPMO-treated rice straw, while a corresponding increase in amount of C2 and C4 was observed. C1 mainly reflects the non-carbohydrate content, such as lignin and extracts (i.e., fatty acids, hydrocarbons) [17]. Extractives contribute most of their signals to C1 [17]. The increase in lignin content, as confirmed by FTIR results, suggests that the decrease in C1 content induced by *An*LPMO is likely attributed to a reduction in the content of extractable materials, such as the wax layer [18]. Sain and Panthapulakkal observed a lower C1 proportion in microbially retted fibers compared to mechanically processed fibers, positing that this discrepancy primarily arose from the removal of extractives [20]. Cellulose, lignin, xylan, and amino groups collectively contribute signals to C2 [17,19]. The observed elevation in lignin content may potentially contribute to the augmentation of the C2 proportion. Furthermore, the *An*LPMO-induced increase in C-N bonds on the surface of rice straw may facilitate an elevation in the C2 proportion, as evidenced by the observed rise in surface nitrogen content as determined by XPS survey. The control group exhibits a low proportion of C4, with only one sample in the control group detecting the presence of C4. The absence of C4 corresponding to the carboxylic ester or acids could potentially be explained by the higher concentration of hydrocarbons and/or extractives (C1) that have accumulated on the surface of the rice straw. These substances may have impeded the detection of C4 carbon atoms through the utilization of the XPS technique [20].

The findings from the initial *in vitro* fermentation experiment indicated that the addition of *An*LPMO enhanced the rumen fermentation of rice straw. However, as *An*LPMO was also a protein in nature, it remained uncertain whether the enhancement in rumen fermentation of rice straw could be attributed to its provision as a nitrogen source to microorganisms, its biological function of disrupting the fibrous structure of the substrate, or a combination of both factors. Consequently, a subsequent *in vitro* fermentation experiment was conducted, wherein the control group was supplemented with inactivated *An*LPMO to mitigate the influence of *An*LPMO as a nitrogen source. The results showed that, akin to the initial fermentation outcomes, the incorporation of *An*LPMO enhanced the rumen fermentation of rice straw. This enhancement was evident in the augmentation of IVDMD, the generation of total VFA, acetate, and propionate, as well as the concentration of ammonia-N. The findings demonstrated that the bioactive function of *An*LPMO did contribute to improving the microbial fermentation of rice straw. However, the disparity lay in the extent to which *An*LPMO enhances the *in vitro* fermentation of rice straw in the two fermentation procedures. In the context where the control group and *An*LPMO group exhibited comparable nitrogen levels, the *An*LPMO treatment resulted in a 9.6% increase in IVDMD, a 9.27 m*M* increase in total VFA production, and a 4.1 m*M* increase in ammonia-N concentration. These values were notably lower than the respective increases of 14.8%, 14.1 m*M*, and 12.6 m*M* observed in the initial fermentation experiment. The findings indicated that *An*LPMO, as a nitrogen source, may also play a certain role in improving the microbial fermentation of rice straw.

In the rumen, the fermentation of substrates is intricately linked to the rumen microbiota. VFAs are generated as the final products of microbial fermentation, with dietary carbohydrates such as cellulose, hemicellulose, pectin, starch, and soluble sugars serving as the primary substrates for fermentation. The findings of this study indicate that the presence of *An*LPMO has a significant impact on the microbial community in the *in vitro* fermentation of rice straw, leading to an increase in microbial species diversity. This conclusion is supported by the results obtained from PCoA, Simpson index, and Shannon index analyses. At the phylum level, *An*LPMO increased the relative abundances of Proteobacteria, Tenericutes, Fusobacteria, and Spirochaetes, but reduced that of Firmicutes. Proteobacteria and Spirochaetes can effectively degrade fibrous substances. A recent study showed that the enhancement of straw fiber degradation rate was achieved by enrichment of Proteobacteria bacteria attached to the straw [21]. Multiple studies have identified Spirochaetes as being associated with the degradation of fiber and production of short-chain fatty acids [22]. At the genus level, *An*LPMO increased *Succinivibrio*, *Rikenellaceae_RC9_gut_group*, *Oribacterium*, *Fusobacterium*, *Treponema_2*, *Desulfovibrio*, and *Prevotellaceae_UCG-003*, which were associated with increased IVDMD or/and VFAs. *Succinivibrio*, a phylum Proteobacteria member, was a predominant contributor to increased IVDMD and production of acetate, propionate, total VFA, and ammonia-N following *An*LPMO supplementation. *Succinivibrio* was known for its higher fiber degrading potential and numerous studies have reported the occurrence of enhanced fiber degradation and ruminal VFA production as a consequence of *Succinivibrio* [23]. *Rikenellaceae_RC9_gut_group*, belonging to phylum Bacteroidetes, are also a well-known fiber-degrading bacterium and has a key role in fiber digestion and rumen fermentation [24]. Though very little is known about the role of *Oribacterium* in the intestinal ecosystem, enhanced fiber degradation by the action of *Oribacterium* was reported or the growth of *Oribacterium* bacteria were stimulated by fiberous diet in the previous studies [25]. *Oribacterium* was also positively correlated with IVDMD and ammonia-N concentration in this study. *Prevotellaceae_UCG-003* belonged to the family Prevotellaceae, which can break down dietary fiber and produce the short chains fatty acids in the gut [26]. The existence of positive associations between *Prevotellaceae_UCG-003* and various ruminal fermentation parameters, including IVDMD, acetate, propionate, and total VFA, have been documented in previous studies [27]. *Desulfovibrio*, a member of the sulfate-reducing bacteria group, has the ability to metabolize lactate and pyruvate into acetate and CO_2_, utilizing the latter as an electron donor for sulfate reduction [28]. Consequently, the observed elevation in *Desulfovibrio* levels was expected to correspond with an increase in acetate production. Limited research has been conducted on the direct degradation of fiber by *Desulfovibrio*, however, a prior report believed that *Desulfovibrio* was capable of deriving benefits from, as well as interacting with, fiber degraders, consequently leading to the development and preservation of its ability to firmly adhere to the fiber [29]. A noteworthy positive correlation between *Desulfovibrio* and *Succinovibrio*, *Rikenellaceae_RC9_gut_group*, *Prevotellaceae_UCG-003* in this study confirmed the aforementioned point. *Streptococcus*, *Prevotella_1*, and *Anaerovibrio* were the predominant bacteria suppressed by *An*LPMO. *Streptococcus*, belonging to the family Streptococcaceae, is a widely recognized starch-utilizing bacterium and produces lactic acid as the major end-product of glucose/starch fermentation [30]. *Streptococcus* typically exhibits a decline in abundance as the intake and digestion of dietary fiber increases [31]. *Prevotella_1*, a member of family Prevotellaceae, is involved mainly in carbohydrate and nitrogen metabolism in the rumen, and produces enzymes for hemicellulose degradation [32]. Several studies have documented a positive association between *Prevotella_1* and fiber degradation [32], while different findings have been reported in other studies. The abundance of *Prevotella_1* initially rose and subsequently declined in response to the escalating dietary physical effective fiber level in the study by Xue et al [33]. These findings indicate that the association between *Prevotella_1* and fiber utilization is variable. *Anaerovibrio* is mainly related to the degradation of lipid and glycerol [34]. The reduction in *Anaerovibrio* caused by *An*LPMO in current research may inhibit the decomposition of lipid substances. At the species level, *An*LPMO increased *F. succinogenes*, *R. albus*, and *R. flavefaciens*, which are three typical fibrolytic bacteria in rumen [35]. *T. bryantii*, *P. bryantii*, and *P. ruminicola* were tremendously increased by *An*LPMO. *T. bryantii* has been demonstrated to have an association with the fibrolytic bacteria present in the rumen and, albeit not possessing any fibrolytic activity, could augment fiber degradation when co-cultured with fibrolytic bacteria [36]. *P. bryantii* and *P. ruminicola* exhibit efficacy in the decomposition of hemicellulose and pectin [37]. In contrast to this, the provision of *An*LPMO exhibited a significant inhibitory effect on *A. lipolytica* and *S. bovis*, thereby aligning with the findings that Anaerovibrio and Streptococcus are indeed susceptible to inhibition by *An*LPMO. Similarly, the greater abundance of *A. lipolytica* associated with lower abundance of ruminal cellulolytic bacteria was observed in a previous report [38]. *S. bovis* is a rapid degrader of starch and a major producer of ruminal lactate [39]. Although *S. bovis* is capable of utilizing the metabolites produced by fibrolytic bacteria to support its growth [40], this study indicated a notable decrease in the abundance of *S. bovis* with increased fibrolytic bacteria. This could potentially be attributed to the competition among various bacterial species for limited nutrients, but additional research is necessary to substantiate this hypothesis. Similarly, in the study by Koike et al [14], the increase in rice straw digestibility was accompanied by increased *R. flavefaciens*, *P. bryantii*, and *P. ruminicola* and decreased *A. lipolytica* and *S. bovis*. The bacteria mentioned above, facilitated by *An*LPMO, are expected to significantly contribute to the improvement of rice straw fiber and dry matte degradation.

## Figures and Tables

**Table 1:** Effects of *An*LPMO on the relative atomic percentages, oxygen-carbon ratio, and carbon peak abundance of rice straw

Items	Control	AnLPMO	SEM	p-value
C (%)	75.17^a^	71.44^b^	0.88	<0.01
O (%)	19.32	20.37	0.53	0.38
N (%)	2.96^b^	7.57^a^	1.05	<0.01
Si (%)	2.55^a^	0.62^b^	0.45	<0.01
O/C	0.26	0.29	0.01	0.16
C1 (%)	72.54^a^	63.49^b^	2.48	0.05
C2 (%)	19.37	25.53	1.84	0.09
C3 (%)	7.58	7.63	0.73	0.98
C4 (%)	0.51^b^	3.35^a^	0.72	0.02

*An*LPMO, *Aspergillus niger* lysing polysaccharide monooxygenase; SEM, standard error of the mean.

a,b Different superscripts letters in the same row indicate significant differences (p≤0.05).

**Table 2:** Effects of *An*LPMO on *in vitro* rumen fermentation of rice straw (Exp. 1)

Items	Supplemental *An*LPMO (U)						SEM	p-value

	0	0.5	0.8	1.1	1.4	2.0		
IVDMD (%)	47.54^d^	48.50^cd^	49.95^c^	51.42^bc^	54.57^a^	52.94^ab^	0.52	<0.01
Total gas production (mL)	60.73^b^	63.45^b^	65.58^ab^	69.23^a^	70.12^a^	70.62^a^	1.60	<0.01
pH	7.20	7.12	7.22	6.94	7.07	7.05	0.08	0.08
Total VFA (m*M*)	84.04^d^	86.34^cd^	87.29^c^	91.21^bc^	94.35^ab^	96.61^a^	4.06	<0.01
Acetate (m*M*)	61.75^c^	63.09^bc^	64.17^bc^	67.29^b^	69.32^a^	71.06^a^	3.34	<0.01
Propionate (m*M*)	16.32^c^	18.12^b^	18.08^b^	18.70^ab^	18.74^ab^	19.10^a^	0.57	<0.01
Butyrate (m*M*)	5.74^ab^	4.90^c^	4.92^c^	5.08^b^	5.67^b^	5.96^a^	0.15	0.02
Acetate/Propionate ratio	3.71^a^	3.41^d^	3.49^cd^	3.52^bcd^	3.62^abc^	3.64^ab^	0.08	<0.01
Ammonia-N (m*M*)	3.42^d^	6.90^c^	8.16^bc^	9.71^bc^	10.62^ab^	13.46^a^	1.60	<0.01

*An*LPMO, *Aspergillus niger* lysing polysaccharide monooxygenase; SEM, standard error of the mean; IVDMD, *in vitro* digestibility of dry matter; VFA, volatile fatty acids.

a–d Different superscripts letters in the same row indicate significant differences (p≤0.05).

**Table 3:** Effects of *An*LPMO on *in vitro* rumen fermentation of rice straw (Exp. 2)

Items	Control	*An*LPMO	SEM	p-value
IVDMD (%)	40.05^b^	43.90^a^	0.941	0.01
Total gas production (mL)	31.5^b^	47.5^a^	4.29	0.04
pH	6.99	7.01	0.033	0.22
Total VFA (m*M*)	82.23^b^	91.50^a^	2.24	<0.01
Acetate (m*M*)	64.38	70.02	1.74	0.10
Propionate (m*M*)	14.42^b^	17.90^a^	0.84	<0.01
Butyrate (m*M*)	3.43	3.58	0.20	0.75
Acetate/propionate ratio	4.48	3.93	0.20	0.18
Ammonia-N (m*M*)	1.30^b^	5.35^a^	0.91	<0.01

*An*LPMO, *Aspergillus niger* lysing polysaccharide monooxygenase; SEM, standard error of the mean; IVDMD, *in vitro* digestibility of dry matter; VFA, volatile fatty acids.

a,b Different superscripts letters in the same row indicate significant differences (p≤0.05).

**Table 4:** Effects of *An*LPMO on populations of microbes (% of total bacterial 16S rDNA) in *in vitro* rumen fermentation liquid of rice straw (Exp. 2)

Items	Control	*An*LPMO	SEM	p-value
*Fibrobacter succinogenes* (×10^−6^)	5.36^b^	16.58^a^	2.73	0.01
*Ruminococcus albus* (×10^−6^)	4.60^b^	9.15^a^	1.19	0.03
*Ruminococcus flavefaciens* (×10^−8^)	0.08^b^	0.42^a^	0.09	0.01
*Anaerovibrio lipolytica* (×10^−2^)	127.85^a^	12.58^b^	26.05	<0.01
*Streptococcus bovis* (×10^−6^)	845.20^a^	0.16^b^	195.04	<0.01
*Treponema bryantii* (×10^−6^)	0.71^b^	566.39^a^	140.08	0.01
*Selenomonas ruminantium* (×10^−2^)	21.24	16.99	3.88	0.64
*Prevotella bryantii* (×10^−8^)	0.52^b^	2,988.19^a^	709.75	<0.01
*Prevotella ruminicola* (×10^−2^)	1.25^b^	29.97^a^	6.83	<0.01
*Ruminobacter amylophilus* (×10^−8^)	43.43	36.78	8.80	0.75

*An*LPMO, *Aspergillus niger* lysing polysaccharide monooxygenase; SEM, standard error of the mean.

a,b Different superscripts letters in the same row indicate significant differences (p≤0.05).

## SUPPLEMENTARY MATERIAL

**Supplementary Material**. *AnLPMO* gene

**Supplementary Figure S1**. Sequence alignment between AnLPMO and reference sequence.

**Supplementary Table S1**. Alpha-diversity indices in control and AnLPMO groups

**Supplementary Figure S2**. LEfSe analysis displaying the ruminal bacteria change between control group and AnLPMO group (LDA≥3.0 and p≤0.05 were shown).

**Supplementary Figure S3**. The co-occurrence network interactions of top 30 abundant bacteria genera. A node symbolizes a particular species, with its size serving as an indicator of the number of connections it possesses, while its color denotes its classification at the phylum level.

**Supplementary Figure S4**. Correlation analysis of bacteriaandin vitrofermentation parameters.
