# Supplementary material for: A lysing polysaccharide monooxygenase from Aspergillus niger effectively facilitated rumen microbial fermentation of rice straw
Source: Anim Biosci. 2024 May 7;37(10):1738–50. doi: 10.5713/ab.24.0026 (PMC11366511; doi:10.5713/ab.24.0026)
Supplement: Supplementary file 1 [file ab-24-0026-Supplementary-AnLPMO.pdf]

AnLPMO gene

ATGAAGACTACCACCTACAGTTTGCTCGCTCTGGCAGCGGCTTCCAAGCTGGCTTCCGCCCACACCACC

GTCCAGGCCGTCTGGATCAACGGCGAGGACCAGGGTCTCGGTAACTCCGCCGATGGCTACATCCGCA

GTCCCCCCAGCAACAGCCCCGTCACCGACGTCACGTCTACCGACATGACCTGCAACGTCAACGGTGAC

CAGGCCGCCTCCAAGACCCTCTCCGTCAAGGCCGGTGATGTTGTCACCTTCGAGTGGCACCACAGCGA

CCGCTCCGACTCCGACGACATCATCGCCTCCTCCCACAAGGGTCCCGTCCAGGTCTACATGGCCCCGAC

GGCCAAGGGCTCCAACGGCAACAACTGGGTCAAGATCGCCGAGGACGGATACCACAAGAGCTCTGA

CGAGTGGGCTACCGACATCCTGATCGCCAACAAGGGCAAGCACAACATCACCGTTCCCGACGTTCCCG

CCGGTAACTACCTCTTCCGCCCTGAGATCATTGCCCTCCACGAGGGTAACCGCGAGGGTGGTGCCCAG

TTCTACATGGAGTGTGTCCAGTTCAAGGTCACCTCCGACGGCTCCAGCGAGCTTCCCTCTGGTGTCTCC

ATCCCCGGCGTCTACACCGCCACTGACCCCGGTATCCTCTTCGACATCTACAACTCCTTCGACAGCTACC

CCATCCCCGGCCCGGATGTCTGGGATGGCTCCAGCTCCGGCTCCAGCTCCGGATCCTCCTCCGCTGCTG

CTGCTGCTACCACCTCTGCTGTCGCTGCTACTACCCCCGCCACCCAGGCCGCCGTTGCGGTCTCTTCCTC

CGCTGCTGCTGTCGTTGAGTCCACCTCTTCCGCCGCCGCTGCTACCACCGAAGCTGCCGCTCCCGTCGT

CAGCCAGCAGGCCACCTCCGCTGTCACCAGCCAGGCCCAGGCCCCCACCACCTTCGCCACCTCTTCTA

AGTCCTCCAAGACTGCCTGCAAGAACAAGACCAAGTCCAAGTCCAAGGTTGCTGCTTCCAGCACTGA

GGCCGTCGTTGCCCCCGCTCCTACTTCCAGCGTCGTCCCTGCTGTCAGTGCCAGCGCTAGCGCTTCCGC

TGGCGGTGTTGCTAAGAAGTACGAGCGCTGCGGTGGTATCAACCACACTGGCCCTACCACTTGCGAGA

GCGGCTCCGTTTGCAAGAAGTGGAACCCTTACTACTACCAGTGCGTTGCGTCTCAGTAA
