# Supplementary material for: A lysing polysaccharide monooxygenase from Aspergillus niger effectively facilitated rumen microbial fermentation of rice straw
Source: Anim Biosci. 2024 May 7;37(10):1738–50. doi: 10.5713/ab.24.0026 (PMC11366511; doi:10.5713/ab.24.0026)
Supplement: Supplementary file 2 [file ab-24-0026-Supplementary-Fig-1.pdf]

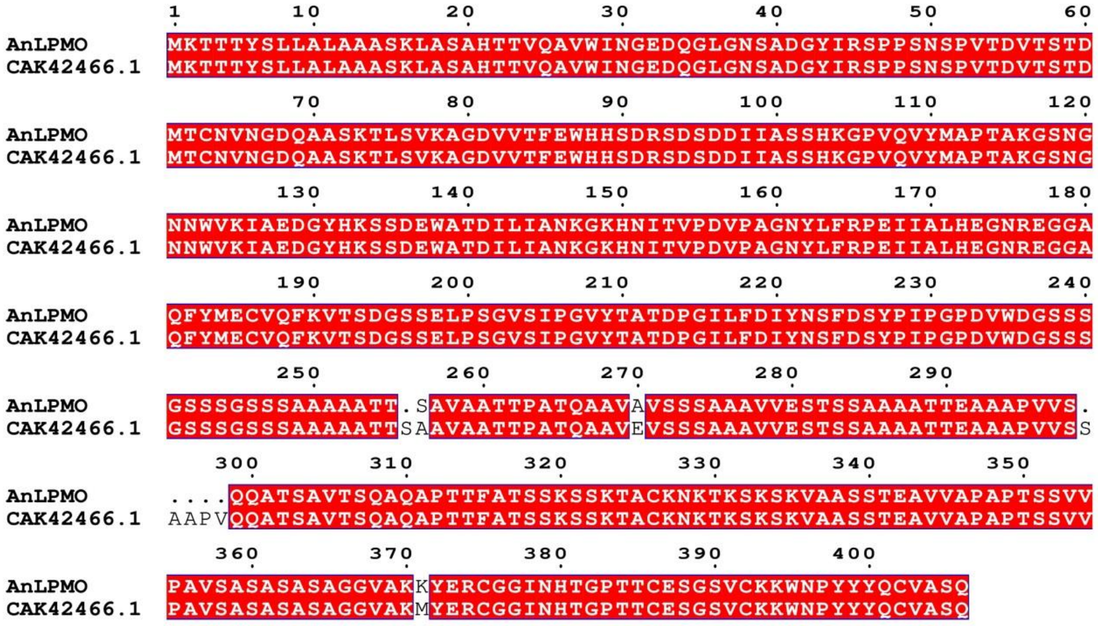

Figure S1. Sequence alignment between *An*LPMO and reference sequence.
