# Supplementary material for: A lysing polysaccharide monooxygenase from Aspergillus niger effectively facilitated rumen microbial fermentation of rice straw
Source: Anim Biosci. 2024 May 7;37(10):1738–50. doi: 10.5713/ab.24.0026 (PMC11366511; doi:10.5713/ab.24.0026)
Supplement: Supplementary file 3 [file ab-24-0026-Supplementary-Table-1.pdf]

Table S1. Alpha-diversity indices in control and *An*LPMO groups

Item	Control	*An*LPMO	SEM	*P*
Chao	372.6	414.6	20.9	0.37
Simpson	0.23	0.13	0.03	0.05
Shannon	3.12	4.28	0.29	0.02
ACE	417.4	454.7	19.4	0.39
Goods coverage	0.999	0.999	0	0.52
