# Supplementary material for: A lysing polysaccharide monooxygenase from Aspergillus niger effectively facilitated rumen microbial fermentation of rice straw
Source: Anim Biosci. 2024 May 7;37(10):1738–50. doi: 10.5713/ab.24.0026 (PMC11366511; doi:10.5713/ab.24.0026)
Supplement: Supplementary file 5 [file ab-24-0026-Supplementary-Fig-3.pdf]

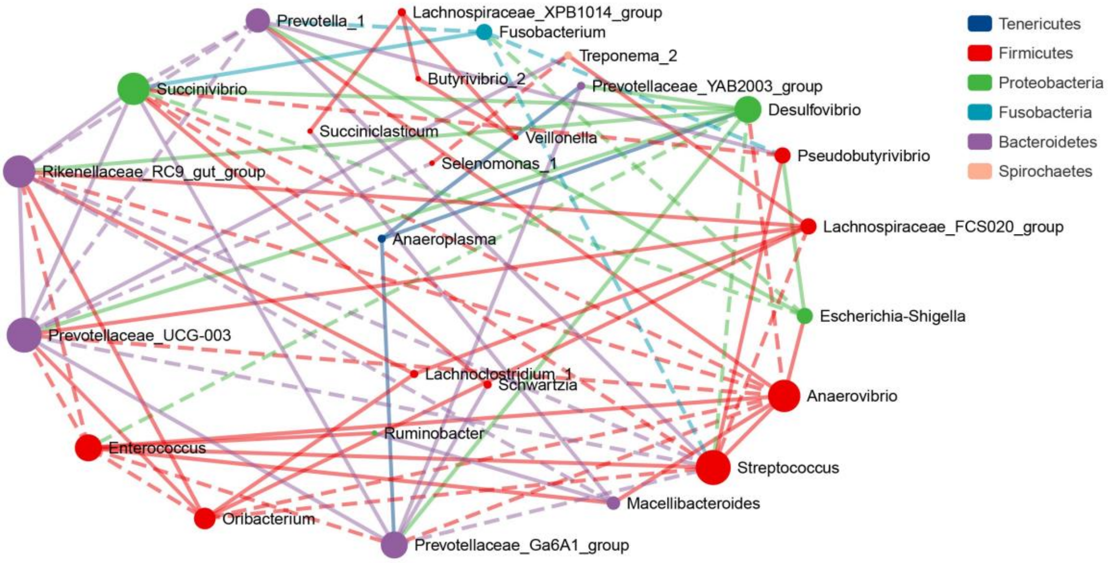


**Figure S3.** The co-occurrence network interactions of top 30 abundant bacteria genera. A node symbolizes a particular species, with its size serving as an indicator of the number of connections it possesses, while its color denotes its classification at the phylum level. The lines connecting these nodes illustrate the relationship between two species, with solid lines denoting a positive correlation, dashed lines indicating a negative correlation, and the thickness of the lines reflecting the magnitude of the correlation coefficient. Only the significant correlations (coefficients $\geq 0.8$ and $P \leq 0.05$) were shown.
