# Supplementary material for: A lysing polysaccharide monooxygenase from Aspergillus niger effectively facilitated rumen microbial fermentation of rice straw
Source: Anim Biosci. 2024 May 7;37(10):1738–50. doi: 10.5713/ab.24.0026 (PMC11366511; doi:10.5713/ab.24.0026)
Supplement: Supplementary file 6 [file ab-24-0026-Supplementary-Fig-4.pdf]

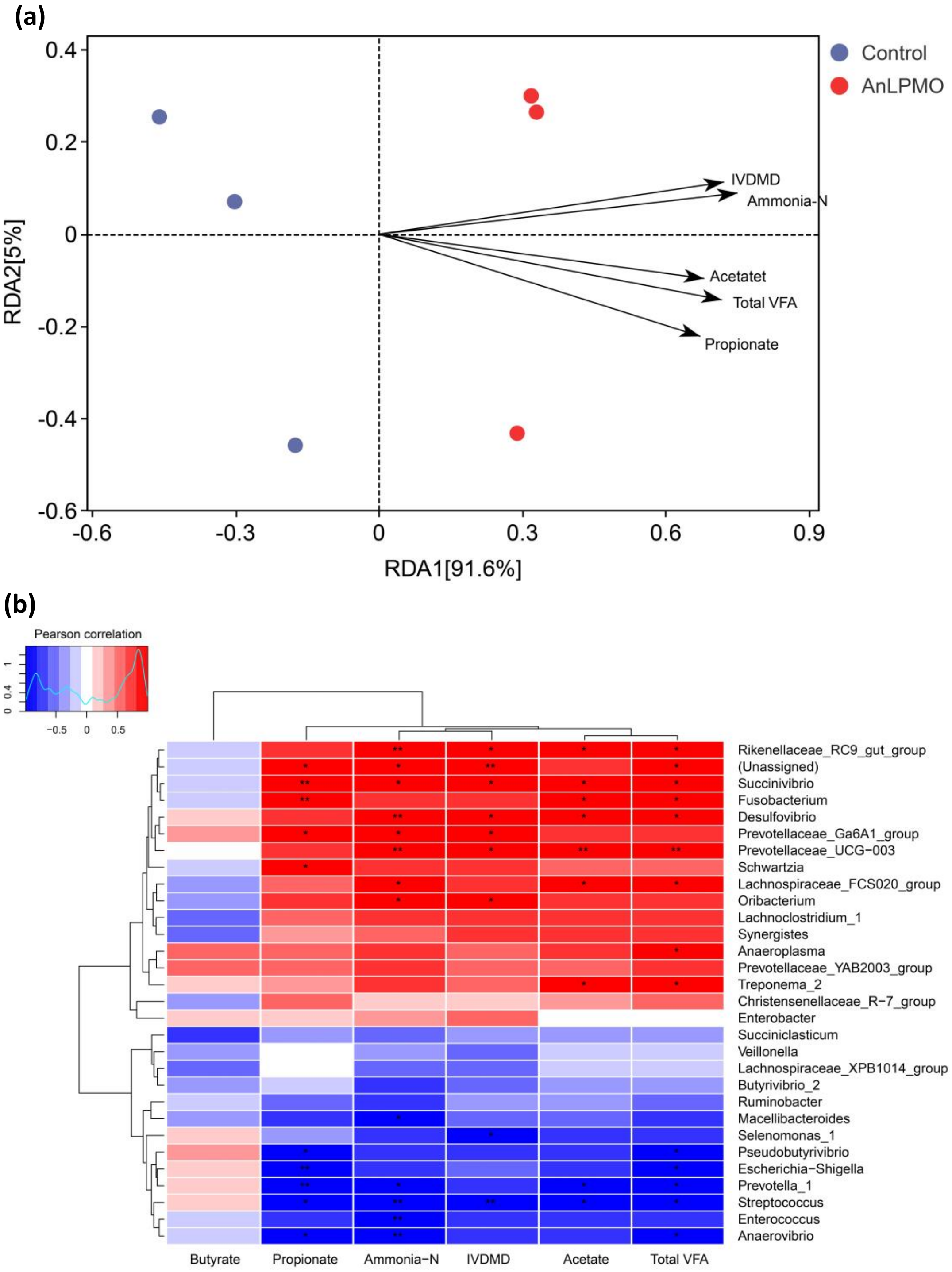

Figure S4. Correlation analysis of bacteria and *in vitro* fermentation parameters. (a) Redundancy analysis of the bacteria composition at genus level relative to *in vitro* fermentation parameters; (b) Heatmap of the Pearson correlations between the 30 most abundant bacterial genus and *in vitro* fermentation parameters. Asterisk indicated the significant correlations (coefficients ≥ 0.8 and $P \leq 0.05$).
